# Preventive Care and Chronic Disease Management Comparison of Appalachian and Non-Appalachian Community Health Centers in the United States

**DOI:** 10.13023/jah.0203.06

**Published:** 2020-07-19

**Authors:** Julie P. Marcinek, Alek Sripipatana, Sue Lin

**Affiliations:** Robert Graham Center for Policy Studies in Primary Care, Washington DC; U.S. Department of Health and Human Services, Health Resources and Services Administration, Bureau of Primary Health Care

**Keywords:** Appalachia, primary care, preventive care, chronic care, clinical quality, underserved population, community health center

## Abstract

**Introduction:**

The Appalachian region is often characterized by poor health outcomes and economic depression. Health centers (HCs) are community-based and patient-directed organizations that deliver comprehensive, culturally competent, high-quality primary healthcare services in high need areas, including Appalachia, where economic, geographic, or cultural factors can hinder access to healthcare services.

**Purpose:**

The study compares the clinical quality performance in preventive care and chronic disease management between Appalachian HCs and their non-Appalachian counterparts.

**Methods:**

Using 2015 Uniform Data System (UDS) health center data, bivariate and multivariate linear regression analyses examine the association of Appalachian HC with performance on preventive and chronic care clinical quality measures (CQMs).

**Results:**

In the multivariate analysis, patients served at Appalachian HCs are more likely to receive colorectal cancer screening and pediatric weight assessment and counseling than at non-Appalachian HCs. No statistically significant differences in performance observed among other CQMs. The percentage of Medicaid patients and total physician FTEs have positive associations with preventive care in colorectal and cervical cancer screening, pediatric weight assessment and counseling, and tobacco screening and cessation intervention as well as chronic disease management of aspirin therapy for ischemic vascular disease and hypertension control in the multivariate model.

**Implications:**

Overall Appalachian HCs perform as well as or better than non-Appalachian HCs in delivering preventive and chronic care services. Further examination of clinical quality improvement programs, insurance payer mix, and practice size among Appalachian HCs could advance the replication of clinical quality success for clinics in similar underserved communities.

## INTRODUCTION

The Appalachian region is a geographically and culturally distinctive region of the U.S. extending from New York to Alabama characterized by largely rural geography and resiliency assets that include strong social cohesion, family ties, social support, and spiritual belief.^1^ Health disparities in the region reflect broader trends in rural America, which include higher prevalence of obesity, diabetes, hypertension, cancer mortality, lower life expectancy, and lower adoption of preventive screening.^2^–^5^ Social–behavioral, economic, and cultural factors associated with Appalachian health disparities include lack of exercise, unhealthy diets, poor health literacy, lack of knowledge about the importance of preventive care, and area-level poverty attributable to high unemployment rates and low family income.^6^ With its rural population, distinctive cultural values, and depressed economic growth, the Appalachian region poses unique challenges in the provision and receipt of preventive and chronic care.^7^

Health centers (HCs) are community-based and patient-directed organizations that deliver comprehensive primary health care services and receive federal grant funding from the Health Resources and Services Administration (HRSA). HCs provide services in high-need areas, including Appalachia, where economic, geographic, or cultural factors hinder access to affordable primary care services. Annually, HCs are required to report clinical quality measures (CQMs) into HRSA’s Uniform Data System (UDS). This study uses the national 2015 UDS data to examine the differences in clinical quality performance in preventive care and chronic disease management between the Appalachian HCs and their non-Appalachian counterparts.

## METHODS

The HRSA’s 2015 UDS data contain health center organizational-level data for patient sociodemographic, services provision, workforce, clinical quality measures, cost, and revenues.^8^ A health center organization may be composed of one or more primary care service delivery sites. This study identifies Appalachian HC as a HC organization with at least one primary care service delivery site located in an Appalachian county, as defined by the Appalachian Regional Commission (ARC).

This cross-sectional study examines six preventive care and three chronic care CQMs reported in the UDS. The preventive measures consist of the following:

1. cervical cancer screening: percentage of women aged 21–64 years who received timely Pap tests to screen for cervical cancer;
2. colorectal cancer (CRC) screening: percentage of patients aged 50 to 75 years who had appropriate screening for colorectal cancer, which could be a colonoscopy within the last 10 years, a flexible sigmoidoscopy within the last 5 years, or an annual fecal occult blood test (FOBT), including the fecal immunochemical (FIT) test;
3. body mass index (BMI) screening and follow-up plan: percentage of patients aged 18 and older with a documented BMI during the most recent visit or within the 6 months prior to that visit and when the BMI is outside of normal parameters, a follow-up plan is documented;
4. weight assessment and counseling for nutrition and physical activity for children and adolescents: percentage of patients aged 3–17 years who had evidence of BMI percentile documentation and who had documentation of counseling for nutrition and who had documentation of counseling for physical activity during the measurement year;
5. tobacco use and screening and cessation intervention: percentage of patients aged 18 years and older who were screened for tobacco use at least once during the measurement year or prior year and who received cessation counseling intervention and/or pharmacotherapy if identified as a tobacco user; and
6. screening for depression and follow-up plan: percentage of patients aged 12 years and older screened for clinical depression using an age appropriate standardized tool and follow-up plan documented.

The chronic disease management clinical quality measures are as follows:

1. aspirin therapy for patients with ischemic vascular disease;
2. blood pressure control (as defined by hypertensive patients with a blood pressure less than 140/90); and
3. rate of uncontrolled diabetes, that is, diabetic patients with a hemoglobin A1c (HbA1c) > 9%.

Bivariate and multivariate linear regression analyses examined the association between being an Appalachian health center and performance on preventive and chronic care clinical quality measures. Other covariates in the regression model include patient characteristics, total number of physician full-time equivalent (FTE) as a proxy for practice size, and patient-centered medical home (PCMH) recognition status. HRSA has supported HC’s implementation of PCMH practice transformation to improve clinical quality through effective care coordination and holistic care. Patient characteristics were composed of percentage of racial/ethnic minority patients, percentage of patients at or below 100% of the federal poverty level (FPL), and percentage of Medicaid patients. Statistical analyses were conducted using SAS version 9.3.

## RESULTS

Table 1 summarizes the demographic analysis of the 147 Appalachian health centers, which represents just over 10% of all HRSA-funded HCs in 2015. The mean gender distribution between the two groups was 42.22% male and 57.78% female in Appalachian HCs versus 43.47% and 56.53% in non-Appalachian HCs (p=0.022). Larger proportion of Appalachian HCs patients were over the age of 65 years (11.54% versus 8.56%). Appalachian HC patient population is comprised of 72.86% non-Hispanic White and 6.14% best served in a language other than English as compared with 38.41% and 19.68% in the non-Appalachian HC patient population respectively (p<0.001). Appalachian HC have higher proportion of patients with private insurance (26.85% vs. 17.93%, p<0.001) and lower proportions of patients with Medicaid (34.96% vs. 44.32%, p<0.001). Appalachian HCs have a lower mean total physician FTE (6.39 vs 8.90, p<0.001). Finally, PCMH recognition percentages are similar for both groups of HCs (51.70% vs 48.29%, p =0.513).

In Table 2, the bivariate analysis of CQMs demonstrates statistically significant differences (p ≤ 0.05) between Appalachian HCs and non-Appalachian HCs for lower performance in cervical cancer screening (46.23% vs. 50.44%) and better performance in blood pressure control (63.66% vs. 61.41%). Neither of the comparisons of the performance among the other five preventive care nor the two chronic disease management CQMs are statistically significant.

In the multivariate linear regression analysis as exhibited in Table 3, Appalachian HC is associated with better performance in preventive care CQMs of colorectal cancer screening and weight assessment and counseling for nutrition and physical activity for children and adolescents. The association between Appalachian HC and cervical cancer screening rates ceases to be statistically significant in the multivariate model. The associations between Appalachian HC and the performance of the other four preventive care CQMs are not statistically significant. For both cervical and colorectal cancer screening, the percentage of Medicaid patients and total physician FTEs have positive relationships. In addition, PCMH recognition is positively associated with cervical cancer screening. We found no statistically significant differences in the chronic disease management CQMs between Appalachian and non-Appalachian HCs. The percentage of minority patients is negatively associated with CQMs for aspirin therapy for ischemic vascular disease and blood pressure control; in contrast, the percentage of Medicaid patients and total physician FTEs have positive associations. Uncontrolled diabetes is positively associated with the percentage of minority patients and patients at or below 100% FPL served; however, there is a negative association with total physician FTEs.

## IMPLICATIONS

Our findings demonstrate that patients receiving health care in Appalachian HCs experience quality of preventive and chronic care on par with patients served at non- Appalachian HCs. In particular, receiving care at Appalachian HCs is not associated with lower cervical cancer screening rate, which suggests Appalachian HCs are making strides towards addressing risk factors associated with receipt of cervical cancer screening such as lack of routine source of medical care, self-image insecurity, cultural stigma, as well as misperceptions about health and cancer.^9^ In a region typically characterized by cultural aversion to health care, poor health care access, and lower participation in preventive health screenings, Appalachian HCs are doing better or comparable at achieving clinical quality performance for underserved patient populations in Appalachian communities.^10^

Of particular interest are findings of positive associations between Appalachian HC and preventive CQMs of CRC screening and pediatric weight assessment and counseling. The CRC screening CQM performance in Appalachia HCs may reflect targeted clinical quality improvement initiatives by HCs and professional organizations to expand CRC screening modalities beyond colonoscopy, address financial barriers, and enhance trust in patient–provider relationships towards screening recommendation adherence.^11^ For example, in West Virginia (the only state that rests completely within the Appalachian region), the state Primary Care Association partnership with the American Cancer Society to advance CRC screening rates resulted in increases in screening rates from 31% in 2013 to 40% in 2015 as well as other federal public health investment in early screening and detection of CRC through outreach to primary care practices in the state. The pediatric weight assessment and counseling finding suggests that the emphasis of patient centered and culturally competent care at HCs has the potential to address the socio–cultural barriers to health screenings.

The primary limitation of this study is that findings from 2015 UDS cross-sectional data do not allow for causal inferences. In addition, the Appalachian HC definition includes any HC with a clinical service delivery site in an Appalachian county, which may potentially lead to the inclusion of primary care service sites in border counties incorporated into CQM data reported at the HC organizational level.

Overall, Appalachian HCs perform better than or comparable to their non-Appalachian counterparts in delivering preventive and chronic care services. Additionally, study findings suggest that HCs may be providing effective primary care that helps offset health care related disparities expected in Appalachia communities. In particular, barriers to Appalachian health disparities can be overcome by culturally competent care delivered in organizations such as HCs that utilize PCMH model of care with strong care coordination and case management to improve CQMs in Appalachian communities.^12^ Future research could explore other factors potentially impacting CQM performance to identify ways of replicating the successful efforts such as diversity of insurance payer mix of Appalachian HCs, rural practice acquisition trends, and the unique governance structure of HC patient majority board of directors, in which HC patients from the communities are providing leadership in addressing community health needs.

**SUMMARY BOX**

**What is already known about the subject?** Health disparities in the Appalachian region reflect broader trends in rural America, which include higher prevalence of obesity, diabetes, hypertension, cancer mortality, lower life expectancy, and lower adoption of preventive screening.

**What is added by this report?** The Appalachian health centers performed better or comparable than their non-Appalachian counterparts in delivering high quality primary care in preventive and chronic disease management.

**What are the implications?** Future research on clinical quality improvement programs, insurance payer mix, and practice size among Appalachian health centers could advance the replication of clinical quality success for clinics in similar underserved communities.

## Figures and Tables

**Figure 1:**
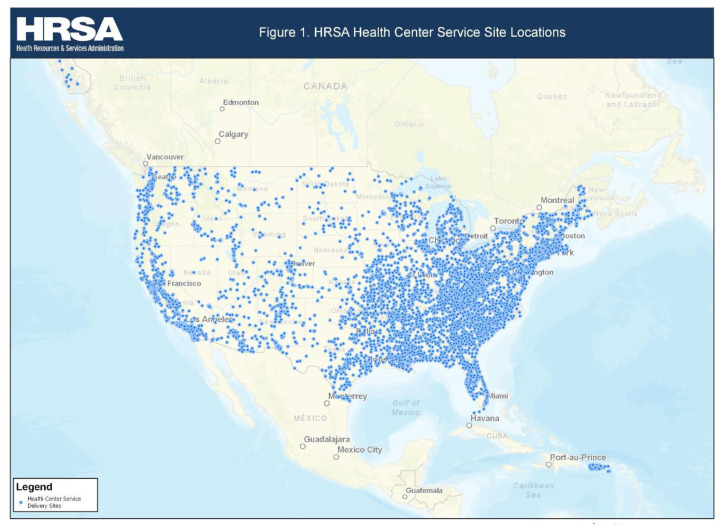
Map of the Locations Of HRSA-Funded HC Primary Care Service Delivery Sites

**Table 1:** Characteristics of Health Centers (HC) and Patients Served by Appalachian Geographic Designation

	Appalachian HCs		Other HCs		
	N=147		N=1228		T-test
Characteristics					p-value
	Mean	SD	Mean	SD	
**Overall**	10.69%		89.30%		
**Gender**					
Male	42.22%	0.061	43.47%	0.072	0.022
Female	57.78%	0.061	56.53%	0.072	0.022
**Race/ethnicity**					
Hispanic	7.43%	0.128	28.28%	0.278	<0.001
Non-Hispanic White	72.86%	0.283	38.41%	0.288	<0.001
Non-Hispanic Black	13.31%	0.203	19.82%	0.242	<0.001
Other	6.40%	0.120	13.48%	0.184	<0.001
**Language Preferred**					
Patients Best Served in a language other than English	6.14%	0.131	19.68%	0.233	<0.001
**Age**					
0–17 Years	23.16%	0.110	26.92%	0.138	<0.001
18–44 Years	36.03%	0.078	37.39%	0.091	0.051
45–64 Years	29.29%	0.065	27.19%	0.089	0.001
65–74 Years	7.39%	0.033	5.62%	0.034	<0.001
75 Years and Older	4.15%	0.026	2.94%	0.027	<0.001
**Household poverty level**					
≤100%	43.95%	0.230	48.75%	0.246	0.025
101–199%	16.73%	0.111	15.69%	0.112	0.290
≥200%	7.15%	0.093	5.87%	0.078	0.110
Not reported	32.18%	0.263	29.69%	0.264	0.282
**Insurance status**					
Uninsured	23.85%	0.190	27.70%	0.192	0.022
Medicaid/CHIP	34.96%	0.149	44.32%	0.202	<0.001
Medicare	14.33%	0.067	9.55%	0.069	<0.001
Private Insurance	26.85%	0.123	17.93%	0.133	<0.001
**Total Physician FTE**	6.37	7.454	8.90	14.544	0.001
	(N, %)		(N, %)		chi-square
**Patient Centered Medical Home Recognition**	76 (51.70%)		593 (48.29%)		0.513

Source: 2015 UDS Data

**Table 2:** Preventive Care and Chronic Disease Management Clinical Quality Measure Performance in Health Centers (HC) by Appalachian Geographic Designation

Clinical Quality Measures	Appalachian HCs (N=147)		Other HCs (N=1228)		T-test
	Mean (%)	SD	Mean (%)	SD	p-value
**Preventive Care Measures**					
1. Cervical Cancer Screening	46.23	0.183	50.44	0.196	0.014
2. Colorectal Cancer Screening	37.79	0.178	34.80	0.193	0.073
3. Adult Body Mass Index (BMI) Screening and Follow-Up Plan	58.55	0.208	55.65	0.230	0.145
4. Weight Assessment & Counseling for Nutrition & Physical Activity (PA) for Children & Adolescents	51.31	0.268	50.44	0.279	0.721
5. Tobacco Use: Screening and Cessation Intervention	77.66	0.216	79.12	0.213	0.434
6. Depression Screening and Follow-Up Plan	51.56	0.272	48.63	0.285	0.236
**Chronic Disease Management Clinical Quality Measure**					
1. Aspirin Therapy for Ischemic Vascular Disease Patients	77.05	0.160	74.98	0.193	0.148
2. Blood Pressure Control (Hypertensive Patients with Blood Pressure < 140/90)	63.66	0.128	61.41	0.133	0.051
3. Uncontrolled Diabetes (Diabetic Patients with HbA1c > 9%)	29.34	0.128	30.97	0.141	0.184

Source: 2015 UDS data

**Table 3:** Multivariate Linear Regression of Preventive Care and Chronic Disease Management Clinical Quality Measure Performance in Health Centers (HCs) by Appalachian Geographic Designation, Part 1

	Cervical Cancer Screening		Colorectal Cancer Screening		Adult BMI Screening & F/Up Plan		Weight Assessment & Counseling for Nutrition & PA for Children & Adolescents		Tobacco Use: Screening & Cessation Intervention		Depression Screening and Follow-Up Plan		Aspirin Therapy for Ischemic Vascular Disease Patients		Blood Pressure Control (Hypertensive Patients with Blood Pressure <140/90)		Uncontrolled Diabetes (Diabetic Patients with HbA1c > 9%)	
	Coefficient	p-value	Coefficient	p-value	Coefficient	p-value	Coefficient	p-value	Coefficient	p-value	Coefficient	p-value	Coefficient	p-value	Coefficient	p-value	Coefficient	p-value
Appalachian HCs	−0.008	0.624	0.037	0.037	0.034	0.097	0.058	0.021	−0.021	0.264	0.026	0.319	0.009	0.569	0.013	0.240	0.007	0.574
% of Minority Patients	0.053	0.005	−0.013	0.488	−0.021	0.363	0.075	0.007	−0.066	0.002	−0.041	0.158	−0.079	<0.001	−0.053	<0.001	0.075	<0.001
% of Patients at or below 100% Federal Poverty Level	0.016	0.459	−0.038	0.091	0.136	<0.001	0.111	0.001	0.060	0.012	0.120	<0.001	0.026	0.209	−0.013	0.367	0.016	0.326
% Medicaid Patients	0.112	<0.001	0.099	<0.001	0.075	0.020	0.170	<0.001	0.107	<0.001	0.044	0.277	0.123	<0.001	0.088	<0.001	−0.009	0.634
Total Physician FTE	0.002	<0.001	0.002	<0.001	0.001	0.109	0.002	0.003	0.001	<0.001	0.001	0.367	0.001	<0.001	0.001	0.001	−0.001	<0.001
Patient Centered Medical Home Recognition	0.030	0.003	0.019	0.059	0.013	0.278	0.021	0.159	0.004	0.702	0.009	0.549	0.010	0.291	0.007	0.283	−0.013	0.086

Source: 2015 UDS Data
